# Gender-biased diagnosis and treatment of depression: considering our blind eye on men’s depression

**DOI:** 10.1186/s12939-025-02569-1

**Published:** 2025-07-01

**Authors:** Andreas Walther

**Affiliations:** 1 Clinical Psychology and Psychotherapy, University of Zurich, Binzmühlestrasse 14, Zurich, 8050 Switzerland; 2 Psychotherapy Research, University of Graz, Graz, Austria

## Abstract

The commentary explores the critical issue of gender bias in the diagnosis and treatment of depression, responding to the study by Bacigalupe et al. [3] that highlights disparities in mental health care for older adults. While acknowledging the study’s strengths, it argues for deeper exploration into systemic biases and gendered symptomatology. Women’s frequent healthcare interactions increase their likelihood of diagnosis and treatment, while men’s reluctance to seek help often results in delayed or missed diagnoses, further complicated by male-typical externalizing symptoms like aggression, risk-taking, and substance abuse. Traditional diagnostic tools and criteria, rooted in prototypical internalizing symptoms, fail to adequately capture these male-typical presentations.

The commentary also underscores the role of traditional masculinity ideologies (TMI) in shaping men’s mental health behaviors. These socially constructed norms promote emotional suppression and self-reliance, exacerbating gender role conflict, dysfunction and discrepancy stress, thereby reducing help-seeking behaviors. High conformity to TMI correlates with poor therapeutic outcomes, higher dropout rates, and diminished treatment efficacy. The commentary critiques the dual bias evident in overmedicalizing women’s mental health while neglecting masculine expressions of distress, advocating for gender-sensitive diagnostic reforms.

In conclusion, the commentary calls for equitable mental health care frameworks that recognize diverse depressive manifestations across genders. Addressing these biases through gender-sensitive practices and diagnostic adjustments can bridge disparities, reduce over- or under-treatment, and foster inclusivity in mental health care systems, ensuring better outcomes for all individuals.

The authors have conducted a valuable study on the potential gender bias in depression diagnosis and antidepressant treatment, offering important insights into disparities in mental health care for older adults [3]. While their methodology and scientific rigor are commendable, a closer examination of the interpretation of their data may be necessary to explore alternative explanations for the observed differences.

The study acknowledges that women have more frequent contact with healthcare services, particularly primary care, which increases their likelihood of being diagnosed and treated for depression [3]. Men, in contrast, are less likely to seek therapy for depression [16, 24] and men’s reluctance to engage with the healthcare system means they are often not diagnosed or get diagnosed only at advanced stages of depressive disorders or after severe crises, such as severe intoxication by alcohol or drugs or suicide attempts [2]. This is corroborated by the fact that the male suicide death rate is four times higher as for women [13] and that men are more than twice as likely to suffer from substance use disorders [12].

Current research increasingly shows that the traditional diagnosis and treatment of depression often does not take sufficient account of the specific gendered aspects and atypical symptoms of men with depression [23]. A large number of studies show that depression symptoms in men often present atypically and often manifest themselves in male-typical externalizing symptoms such as anger, aggression, reduced impulse control, risky behavior, somatization and substance abuse [4, 10, 11, 20, 22, 26, 30, 33, 36]. These symptoms are in contrast to internalizing depression symptoms such as depressive mood, anhedonia, fatigue, feelings of excessive guilt or worthlessness or suicidal thoughts, which reflect prototypical symptoms of depression and are therefore better represented in classic diagnostic instruments as highlighted by Bacigalupe et al. and others [1, 15].

An important aspect of understanding depression in men are traditional masculinity ideologies (TMI), which are socially constructed and accepted standards, norms and beliefs about how boys and men should be and behave [17]. TMI include the following areas in which men should conform to certain behavioral norms: emotional control, self-reliance, pursuit of status, risk-taking, and heterosexual self-presentation, prioritizing work in life, winning, readiness to and emphasis of heterosexual activity (playboy behavior), power over women, and a willingness to resolve conflicts with violence [14, 18, 19]. Strong conformity to these norms is associated with an increased risk of depression [9, 28, 29, 37], suicide attempts and death by suicide [5, 6, 9]; Walther, Grub, et al., [34], and alcohol use disorders [28, 38]. Similar findings on the role of hegemonic masculinity ideals in men’s internalizing and externalizing symptoms — and on measurement and diagnostic biases in men’s mental health — have also been highlighted [31, 32].

TMI often make it difficult for men to seek help for psychological distress and lead to male gender role conflict [7, 8, 21, 37]. Male gender role conflict describes psychological distress that arises when boys or men try to conform to TMI that are often difficult to reconcile with individual needs and values. Discrepancy stress arises when there is a strong endorsement of TMI and at the same time the man cannot achieve the idealized image of his own gender role, which often leads to self-doubt and reduced self-esteem [17]. Men in particular experience discrepancy stress when they believe they do not meet the demands of masculine strength and control, which often leads to shame and insecurity. Dysfunction stress occurs when conformity to TMI has negative consequences. In this case, strict adherence to TMI, such as avoiding emotions or inflexibly showing dominance, leads to problems such as social isolation, aggression or poor interpersonal relationships [17]. Regarding psychotherapy, recent studies indicate that high conformity to TMI and the experience of gender role conflict may lead to therapy interfering processes in psychotherapy (Walther, Ehlert, et al., [35]). For example, men with high conformity to TMI are more likely to drop out from psychotherapy [27] and report lower psychotherapy outcome expectations [25] than men with low conformity to TMI.

By juxtaposing the SNAC-K-based diagnosis—a gender-blind measure of depression—with register-based diagnoses, the highlighted article by Bacigalupe et al. infer systemic biases that might stem from both diagnostic instruments and clinical practices. I, however, argue, that men’s reluctance to seek mental health care for depression and the presentation of male-typical externalizing depression symptoms are relevant factors keeping depression diagnosis and antidepressant treatment unnecessary low, exacerbating disparities by pathologizing feminine expressions of distress while neglecting masculine ones.

Relating this back to Bacigalupe et al.‘s conclusions, the gender disparities in depression diagnosis and treatment seem to reflect a dual bias: overmedicalization of women’s mental health and a blind eye of gendered symptom presentation in depression in men. This study calls for gender-sensitive reforms in diagnostic criteria and clinical practices, aiming for a more equitable mental health care framework that acknowledges the diverse manifestations of depression across genders. By addressing these biases, healthcare systems can better support both men, women and individuals of other genders, reducing the risk of over- or under-treatment and fostering more inclusive mental health care for all.

## Data Availability

No datasets were generated or analysed during the current study.
